# *MiSNPDb*: a web-based genomic resources of tropical ecology fruit mango (*Mangifera indica* L.) for phylogeography and varietal differentiation

**DOI:** 10.1038/s41598-017-14998-2

**Published:** 2017-11-02

**Authors:** M. A. Iquebal, Sarika Jaiswal, Ajay Kumar Mahato, Pawan K. Jayaswal, U. B. Angadi, Neeraj Kumar, Nimisha Sharma, Anand K. Singh, Manish Srivastav, Jai Prakash, S. K. Singh, Kasim Khan, Rupesh K. Mishra, Shailendra Rajan, Anju Bajpai, B. S. Sandhya, Puttaraju Nischita, K. V. Ravishankar, M. R. Dinesh, Anil Rai, Dinesh Kumar, Tilak R. Sharma, Nagendra K. Singh

**Affiliations:** 1 Centre for Agricultural Bioinformatics, ICAR-IASRI, New Delhi, India; 2 ICAR-National Research Centre on Plant Biotechnology, New Delhi, India; 3 ICAR-Indian Agricultural Research Institute, New Delhi, India; 4 ICAR-Central Institute for Subtropical Horticulture, Lucknow, India; 5 ICAR-Indian Institute of Horticultural Research, Bengaluru, India

## Abstract

Mango is one of the most important fruits of tropical ecological region of the world, well known for its nutritive value, aroma and taste. Its world production is >45MT worth >200 billion US dollars. Genomic resources are required for improvement in productivity and management of mango germplasm. There is no web-based genomic resources available for mango. Hence rapid and cost-effective high throughput putative marker discovery is required to develop such resources. RAD-based marker discovery can cater this urgent need till whole genome sequence of mango becomes available. Using a panel of 84 mango varieties, a total of 28.6 Gb data was generated by ddRAD-Seq approach on Illumina HiSeq 2000 platform. A total of 1.25 million SNPs were discovered. Phylogenetic tree using 749 common SNPs across these varieties revealed three major lineages which was compared with geographical locations. A web genomic resources *MiSNPDb*, available at http://webtom.cabgrid.res.in/mangosnps/ is based on *3-tier architecture*, developed using PHP, MySQL and Javascript. This web genomic resources can be of immense use in the development of high density linkage map, QTL discovery, varietal differentiation, traceability, genome finishing and SNP chip development for future GWAS in genomic selection program. We report here world’s first web-based genomic resources for genetic improvement and germplasm management of mango.

## Introduction

Mango (*Mangifera indica* L.) a member of order *Sapindales*, family *Anacardiaceae*, is one of the popular fruit crops of tropical and sub-tropical ecological regions of the world. Especially in Asia, it is also referred as ‘King of fruits’ of the tropical world^1^. Its fruit is globally known for the strong aroma, coloration, delicious taste and a high nutritive value due to high content of vitamin C, β carotene and minerals^2^. Apart from being used as a fruit, it is also used in various processed forms like pickles, chutney, jelly, vegetable dishes and mango shake. It ranks as the fifth most produced fruit crop worldwide after banana, grapes, apples and oranges. India ranks first in the world in mango production with an annual production of 18–19 MT from an area of 2.31 MHa, which constitutes 40% of the total world production (>45MT worth >200 billion US dollars) (FAOSTAT-2014). India has exported more than 40000 ton mangoes majorly to UAE, UK, Saudi Arabia and the USA, which was of worth 50 million US dollar in 2013–14.

*Mangifera* species being ecologically sensitive, it is mostly distributed below 300 m but can occur at 600–1900 m above sea level. The species is found as scattered individuals in tropical lowland rain forests on well-drained soil. Mango requires a frost-free climate. Flowers and small fruit can be killed if the temperature drops below 40° F, even for a short period. It requires warm and dry weather to set fruit. India is bestowed with substantial soil diversity with as much as 24 soil types^3^ and 15 broad agro-climatic zone having 127 sub agro-climatic zones^4^. The ecological diversity of soil and climate of India has resulted in very high diversity in mango germplasm along with its uniqueness in taste, aroma and colour specific to geographical locations. Though India is having more than thousand varieties of mango, about 30 varieties are grown for commercial purpose^5^. Though the area under mango cultivation has increased substantially over the period, but there has been a meagre increase in yield. Biotic (mainly mango malformation due to *Fusarium mangiferae*)^6^ and abiotic (temperature, humidity, light, drought, soil salinity and nutrient) factors had been major limiting factors in sustainable productivity of mango^7^. Though mango holds a huge economic aspect but owing to lack of genomic resources, genomic-based trait improvement and management has been one of the major impediments in mango productivity.

Mango is an allotetraploid (2n = 40) fruit tree with a small genome size of 450Mb^8–10^ but its whole genome sequence is yet to be completed. Single nucleotide polymorphisms (SNPs) are the most abundant type of genetic markers and their high abundance in the genome makes them the ideal markers to study the inheritance of genomic regions^11–13^. Limited genic region SNP from transcriptome data of mango tissues have been reported^14^ but the whole genome-based bulk SNP discovery is yet to be reported. Genic region SNPs has been tried for varietal differentiation but with very limited success^15^. SSR marker-based approach has also been reported with limited success for example out of 41 only 5 varieties^16^, out of 36 only 7 varieties^17^ could be differentiated. These studies indicate that existing markers are not good enough for differentiation of mango varieties and there is need to discover more number of markers for variety differentiation and mapping^18^. An attempt has been made to develop genomic resources using NGS technology, but these resources are neither accessible to global community, nor it covers different varieties representing gene pool^19^.

RAD-Seq (restriction site associated DNA sequencing) approach can identify thousands of SNPs distributed randomly across the genome^20^. RAD-Seq can be applied to study the population genetics of a species with no or very less sequence data and has several advantages over other methods for SNP discovery. Such approach reduces the complexity of the genome by sub-sampling only at the sites identified by restriction enzymes^21^. It reduces the investment drastically^22^. Till whole genome sequencing of Mango genome is completed such RAD-based bulk SNP discovery approach is most cost-effective and readily feasible alternative. Such SNP can directly be used for phylogeography and varietal differentiation. Similar studies have been done very successfully in various crops like cotton^23^ and sunflower^24^. Besides this such SNP data has further immense use and relevance in increasing marker density in linkage mapping programme. Even such approach has increased the marker density in specific variety of rice by utilizing available ref-sequence^25^. High heterozygosity in the plant is a big challenge in terms of whole genome sequencing-based SNP/polymorphism discovery.  The RAD-seq technique can be used for rapid marker discovery and genotyping in crops for highly heterozygous and outbreeding species like mango where *de novo* genome assembly is challenging^26^.

Attempts of geographical and varietal delineation of mango varieties with their genetic relationship have been made with RAPD and ISSR markers but there is no clear report covering the entire geographical locations of India^27^. Unless markers from all important varieties of mango are not discovered such studies cannot be completed. Molecular markers are required for traceability of mango fruit and its product to address the issues of adulteration^28^. An allelic database of mango for varietal differentiation has been reported^29^ where limited markers are not able to differentiate varieties. Earlier reported molecular marker information are not in the form of web genomic resources to be used as a research tool for mapping, QTL studies and genome finishing. Moreover,  mango being fruit crop of relatively much longer life cycle (2–4 years), the DUS test statutory norms of varietal identification having two trials at two locations (http://www.plantauthority.gov.in/pdf/mango.pdf) becomes further challenging and cumbersome. The available genomic resources in mango are very scarce as only 107768 nucleotide sequences of mango are available in NCBI Genbank (as in May, 2017). Thus for a crop like mango, more holistic genomic resources are required to accelerate the DNA-based varietal differentiation, phylogeographic studies, increase in marker density for subsequent QTL and gene discovery.

Present work aims at ddRAD-based SNP mining from 84 varieties of mango along with the development of its web-based genomic resources. It also aims at phylogeographic studies of these mango varieties along with its evolutionary relationship.

## Results and Discussion

After pre-processing of RAD-Seq data, a total of 171807860 paired end reads were obtained with 14121079231 number of bases. Approximately, 5587436 (3.14%) low quality reads were removed. A total of 1258705 SNPs were obtained from these RAD sequences representing 84 varieties of mango. RAD genomic data coverage of each variety are shown in Supplementary Table 1. Variety-wise average genome coverage was found to be 8.36% with highest coverage of 15.35% in Langra variety and lowest coverage of 3.53% in Ratoul. Average SNP depth was found to be 9.815 with lowest and highest densities 4.787 and 17.953 in varieties, *viz*., Ratoul and Suvarnarekha, respectively. Conversely, in the data set, highest (at 1480 bp interval) and lowest (at 5090 bp interval) SNP density was observed in Suvarnarekha and Ratoul varieties, respectively. Overall SNP density over entire genome was estimated to be 357 bp. All these data have been populated in *MiSNPDb* to develop genomic resources. Only 749 SNPs were found common to all 84 varieties, which were subsequently used for diversity analysis and phylogeography studies. With increase in number of varieties for SNP discovery, we found decrease in number of common SNPs. For example, comparison of 10, 20, 50, 70 varieties, we got 10634, 7253, 2062 and 1343 SNPs, respectively (Table 1). Thus, large number (84) of varieties having inherent limitation of RAD-sequence data variation in terms of area covered on the respective genome of each variety made them further limiting the common genomic regions to be represented in SNP discovery. Besides this, the inherent limitation of RAD sequencing itself has also contributed in this reduced number of common SNPs. Unlike whole genome sequencing, in RAD sequencing, the entire genome cannot be covered irrespective of increase in depth as size selection step discards unsheared or partially sheared restriction fragments^30^. This random missing of genomic region further limits the common regions to be compared in SNP discovery. It leads to reduction in common number of SNPs. Apart from these two general limitations of RAD, there is one species specific reason in our dataset reducing number of common SNPs. Mango species being highly heterozygous causes hindrance in genome assembly, thus compromising genomic coverage of final RAD assembly^31^. All these factors might have contributed in drastic reduction of common number of SNPs in our dataset.

**Table 1:** Decreasing trend in number of common SNPs with increasing number of varieties.

Number of Varieties	Number of Common SNPs
5	32170
10	10634
20	7253
30	4080
40	2184
50	2062
60	1489
70	1343
80	946

Magnitude of reduction in common SNP may not affect the phylogenetic tree, varietal differentiation, admixture and pedigree analysis. In crop like cassava, even 300 SNP markers have been found adequate enough for these kind of differentiation^32^. Such use of RAD based SNP in 429 wheat variety differentiation using just 43 SNPs has been reported^33^.

### SNP-based diversity analysis

SNP-based diversity analysis of the 84 varieties of mango was done along with construction of phylogenetic tree for geographical relatedness using common RAD loci. The major allele frequency varied from 0.5 to 0.994 while minor allele frequency ranged from 0.006 to 0.5. The observed heterozygosity had a range of 0.0579 to 0.2649 with a mean of 0.1246 and expected heterozygosity varied from 0.029 to 0.1324 with a mean of 0.0623. The observed homozygosity was from 0.7351 to 0.9421 with a mean of 0.8753 and expected homozygosity varied from 0.8676 to 0.971 with a mean of 0.9376. The nucleotide diversity (π) had a range of 0.0579 to 0.2649 with a mean of 0.1246. The summary statistics for all the population of mango are summarized in Supplementary Table 2. This table contains summary of genetic statistics for all population split into those calculated for only nucleotide positions that are polymorphic in at least one mango population (described at top, “Variant positions”), as well as all nucleotide positions across all RAD sites regardless of whether they are polymorphic or fixed (described at bottom, “All positions”). These statistics include the average number of individuals genotyped at each locus (N), the number of variable sites unique to each population (private), the number of polymorphic (top) or total (bottom) nucleotide sites across the data set (sites), percentage of polymorphic loci (% poly), the average frequency of the major allele (P), the average observed heterozygosity per locus (H_obs_), the average nucleotide diversity (π), and the average Wright’s inbreeding coefficient (F_IS_).

A total of transition type SNPs (857317, 68.11%) having A/G (427968) and C/T (429349) type, accounting for 34.00% and 34.11%, respectively were detected. The other four types were transversion type (401388, 31.88%), observed as C/G (83914), G/T (100260), A/C (98811) and A/T (118403) with percentage varying between 6.66% and 9.40% (Table 2). The transition to transversion (Ti/Tv) ratio was observed to be 4.27. Ti/Tv is a ratio representing rate of single nucleotide change and not of observed events. Since transitions are two times more frequent than transversions, the Ti/Tv ratio is twice the ratio of events = 2 × (Ti/Tv)^34^. The Ti/Tv ratio approximately nears the Ts/Tv estimated in non-long terminal repeat (Non-LTR) retrotransposon sequences in crops like maize (3.9), medicago (3.6), lotus (2.5)^35^, peach (3.62, 3.47 and 3.28)^34^ and in another case of peach genotype (3.098)^36^. This high transition/transversion ratio of 4.312 in this current study may be an indication of low genetic divergence as it has been noted that lower is the genetic divergence, higher is the Ti/Tv ratio^37^. The Ti/Tv ratio is commonly used for ‘phylogentic’ tree construction, genetic divergence time estimation and to better the mechanisms of evolution at molecular level^38,39^. The number of SNP per variety of mango has been shown in Table 3.

**Table 2:** Statistics for the identified SNP types.

Type of Variation	Number	Percentage of the SNP type
A/G	427968	34.00%
C/T	429349	34.11%
A/T	118403	9.40%
A/C	98811	7.85%
C/G	83914	6.66%
G/T	100260	7.96%
**Total**	1258705	100%

**Table 3:** Number of SNPs detected in each of the 84 varieties of mango.

Variety	Number of SNPs	Variety	Number of SNPs	Variety	Number of SNPs
Afeam	17749	Fazri Kalam	11889	Mohanbhog	8980
Alphan	16439	Gilas	8132	Mombosa	18388
Alphanso	12305	Gola Bhadaiya	19057	Mulgoa	18270
Amin Prince	13929	Gourjeet	9891	Mundappa Black	8955
Amrapali	14002	Gulab Khas Green	11236	Neelum	15352
Arka Aruna	18636	Hardil Aziz	10045	Nekkare	16946
Arunika	8913	Heraswania	26619	Prabhashankar	5018
Baganapalli	19227	Himsagar	31479	Primor de Amoreira	9979
Banganpalli	16458	Hyb. 165	13375	Pusa Arunima	22087
Banglora	13851	Irwin	9889	Pusa Lalima	16964
Baramasi	19290	Iturba	18228	Pusa Peetamber	13154
Baramasi Ajholi	13286	Janardan Pasand	15850	Pusa Pratibha	11330
Bathui	11030	Kala	13867	Pusa Shersth	12922
Bhadaiya Sukul	15080	Kalapahar	8873	Pusa Surya	14123
Bhadauran	11122	Karishad	9704	Ramkela	16380
Bombay	7440	Kesar	19130	Rataul	17558
Bombay Green	13236	Khasulkhas	14202	Ratna	14419
Bombay Yellow	9793	Kothapalli Kobbari	13768	Rosari	16491
Bride of Russia	22978	Kurukkan	11571	Safdar Pasand	14382
Carabao	15576	Langra	30131	Samar Bahist Alibagh	7946
Chandrakaran	13538	Langra Gorakhpur	14260	Seipia	17643
Chinku	12245	Machhli	15548	Sensation	13110
Creeping II	15272	Malda	15530	Sonatol	9698
Dushehari	15095	Malihabad Safeda	13653	Sukul	18788
Edward	13431	Mallika	19592	Suvarnarekha	37688
Elaichi	9243	Manipur dwarf	11064	Tatoul	3122
Extrema	15042	Manorajan	33248	Willard	26827
Fazri	13350	Mohammada Vikarabad	19862	Zardalu	4936

### SNP-based phylogeography of mango varieties

**Figure 1:**
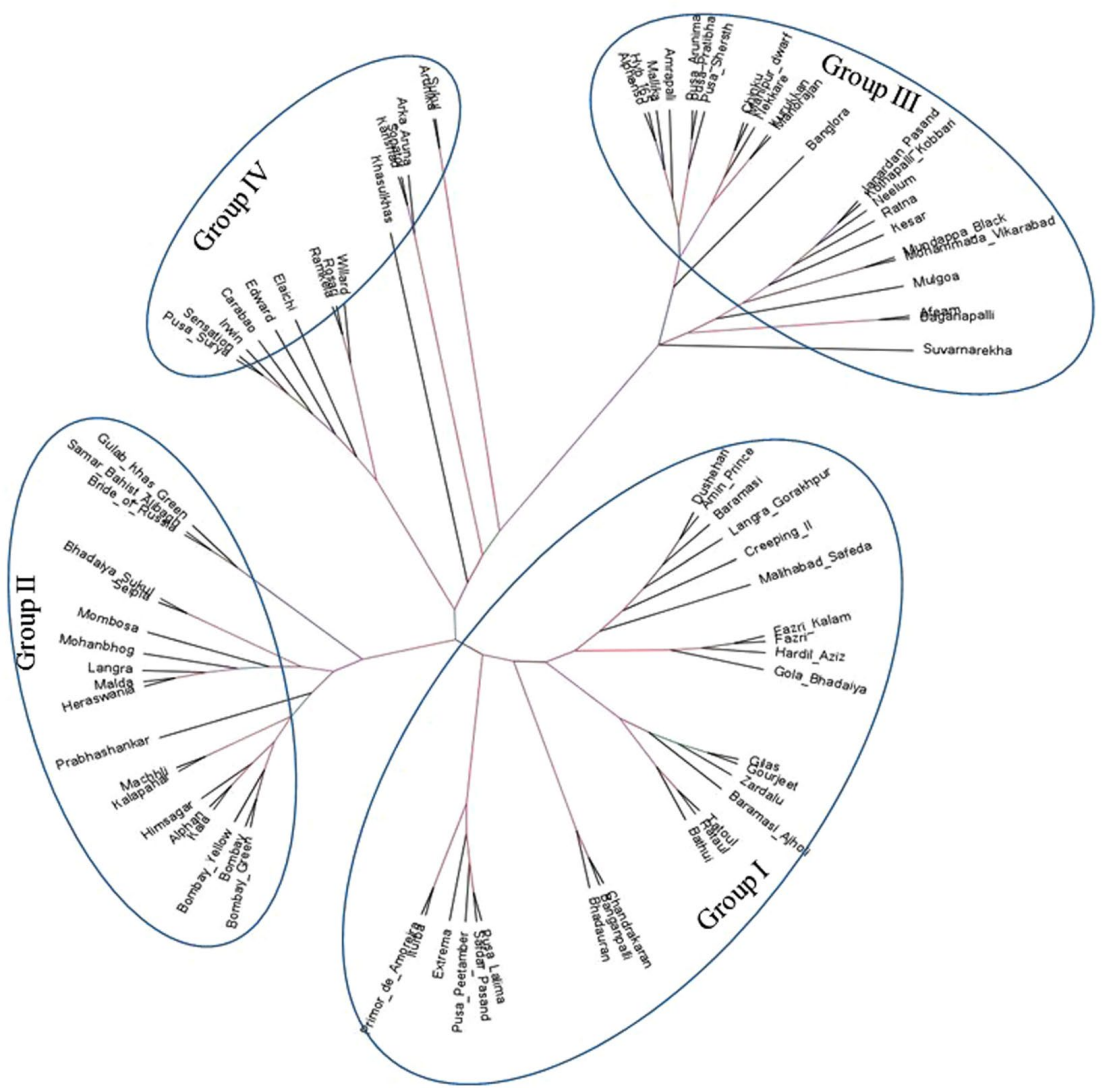
Radial phylogenetic tree showing four major lineages and one minor lineage using the SNP data generated from dd-RAD sequencing information of 84 mango varieties.

The phylogenetic tree of 84 varieties of mango was constructed using PHyML in order to classify the varieties according to their geographical relatedness. The phylogenetic tree constructed from the phylip file of these common RAD loci are shown in Fig. 1. When we compared varieties in this phylogenetic tree with their respective geographical distribution in India, we found all 70 indigenous mango varieties to fall in three zones, *viz*., North, East and South zones except 13 hybrid/exotic varieties. Our analysis revealed three major lineages which showed that varieties belonging to northern and eastern India were found to be overlapping (merged) in geographical distribution.

As our study reveals distinct genetic differentiation in consonance with geographical distribution, thus these SNPs can be used in varietal differentiation of indigenous mango varieties along with traceability of mango produce/products. Hybrid/exotic varieties (13) taken under present study could not be assigned to any of these major lineage/group over three geographical zones. This is obviously expected due to attribute of a hybrid having been originated from two parental varieties. This unassigned group also includes Indian mango varieties having exotic in origin/exotic parental variety (Supplementary Table 3). Small, separate cluster of these hybrid/exotic mango varieties might also be due to the limitation of RAD-based SNPs itself due to restriction fragment bias, restriction site heterozygosity and PCR GC content bias^30^.

Earlier attempt has been made for phylogenetic studies of Indian mango varieties using SSR markers only. SSR-based phylogenetic tree of 387 mango accessions using limited 14 SSR loci has been reported^40^. The present study has 70 common varieties and all agree with earlier reported SSR-based tree, except 14 hybrid/exotic mango varieties. When our 84 varieties were compared for its geographical distribution, we found appropriate grouping in phylogenetic clusters except for hybrid/exotic varieties (Supplementary Table 3). We also compared our SNP-based tree with another SSR-based tree, which is confined to a very limited number of varieties (37) with 14 SSR markers^41^. This study also revealed three major clusters of Indian mango varieties. Subsequent comparison with major commercial mango varieties reported in this study was found in matching clusters^41^.

### Development of web-based genomic resource

**Figure 2:**
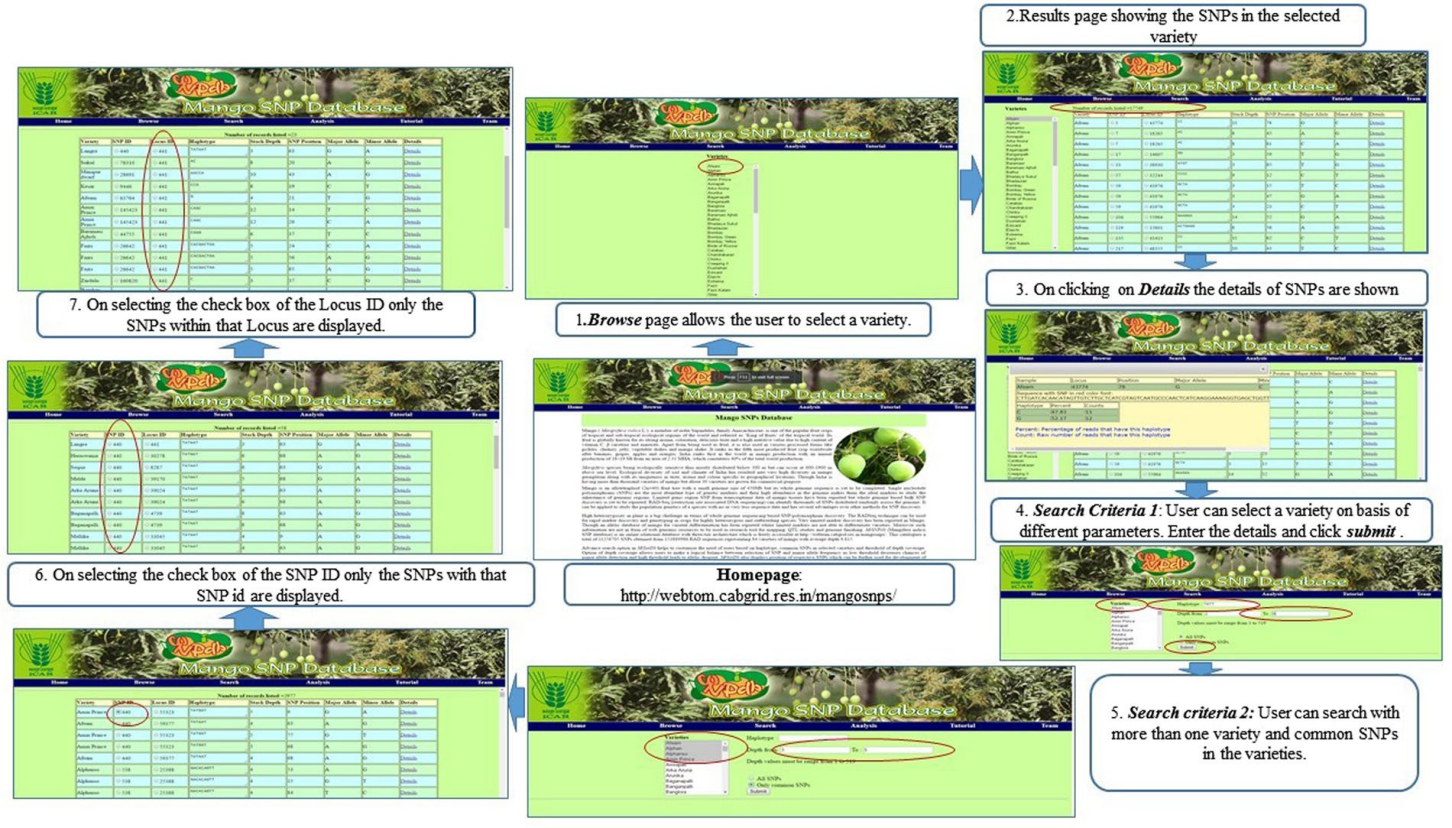
Workflow of the *MiSNPDb* genomic resource.

Though a huge catalogue of phenotypic information of germplasm accessions and related basic information are available in mango resources information system (www.mangifera.org) but there is no web-based genomic resources having molecular markers with genome information. The SNP data sets generated in the present study was compiled and stored in a database (*MiSNPDb*), which is the first portal available with basic information of genomic information. In the homepage of the web portal, selection of radio button ‘variety’ displays total number of SNPs discovered in a particular variety. On clicking ‘SNP ID’ which is having assigned a serial number of every SNP across varieties (from 1 to 1.258705 Million), it will enlist varieties having very same SNP along with type of haplotype with respect to varieties. Our database also displays the stack depth for every SNP so that user can select SNP at desired threshold of stack depth to maneuver over confidence limit required in SNP array assay development. An advance search option has also been provided to customize the need of users based on haplotype, common SNPs in selected varieties, the threshold of depth coverage (Fig. 2). The option of depth coverage will allow users to make a logical balance between selection of SNP and minor allele frequency as low threshold decreases chances of minor allele detection and high threshold leads to allelic dropout^42^. Our advance search has provision for haplotype, depth selection and selection of multiple varieties to get their common SNPs.

### Utility of *MiSNPDb* in genomic research

Present database can be a valuable tool in expediting mango genomic research. As whole genome assembly of mango is yet to be available, thus such RAD-based genomic resources can be a rapid and cost effective approach for marker discovery which can be used in both model and non-model species^43^.

Our genomic resources of RAD data based SNPs can be used in making high-density linkage map of mango. First high density linkage map of mango has been reported by crossing two varieties *viz*., “Jin-Hwang” and “Irwin” of *M. indica*, with length map 3148.28 cM, 20 linkage group and average markers distance 0.48 cM but these resources are also not accessible^44^. Another linkage group (20) of mango genome which are mapped using only genic region 1054 SNPs, thus representing very limited part of the genome^14^. Such use has been reported in other crop like Jujube (*Ziziphus* species) where 42784 putative high-quality SNP were linked to genetic map reducing the average marker interval of very drastically in tune of 0.34 cM making consistency with 12 linkage group i.e. haploid chromosome number^45^. Similar mapping and QTL discovery using RAD-based SNPs have been reported in barley as well^46^. In case of crop sesame, more number of RAD-based SNP has been added in the existing linkage map, to create ultra-dense genetic map or ‘improved SNP map’. This approach has discovered more number of QTLs associated with economically relevant traits with identification of candidate genes in sesame^47^.

RAD data-based genomic resources of SNP markers has enormous application in genome assembly especially to assign scaffolds in process of genome finishing^48^. Such RAD-based SNPs have been successfully used in improvement of *de novo* genome assembly^46^.

These mango SNPs can be used in the development of throughput SNP arrays (SNP chip) after using standard filtering criteria. Such approach and criteria have been reported in other crop like sunflower^24^. MAF information is directly relevant to selection of SNPs for array development in genomic selection program. Mango being highly heterozygous fruit crop with higher genome complexity^27^, thus RAD-based SNP can reduce the magnitude of this issue as this approach is covering on an average 10% of whole genome randomly over specific orthologous regions^49^. Further, such resources have been used in trait specific mapping in very same sunflower^50^. RAD-based SNPs have been successfully used in eggplant for increasing the marker density and development of SNP chip/array^51^.

Mango fruit traceability studies have been reported with a limited number of markers (*i.e*., 15) and limited varieties (41) differentiating Iranian varieties originated from India and Pakistan^16^. Since such differentiation among these disputed varieties requires genotyping of few selected SNPs, thus present *MiSNPDb* can be of immense utility as immediately available genomic resources. Respective location of SNPs displayed in our database along with the DNA fragments can directly be used in development of SNP allelic discrimination chemistry or screening assay like FRET, beacon, scorpion or multiplexing.

DNA profiling provides novel approaches to varietal identification having advantage over traditional morphological comparisons of DUS features. Such advantages are due to the attributes of high resolving power, objectivity, and its feasibility of testing at any stage of plant development/from any tissue of plant besides cost effectiveness^52^. Our RAD SNP-based phylogenetic tree was superimposed over native specific geographical regions of major commercial mango varieties. It clearly reflects that this genomic resources can be used for not only varietal differentiation but also for geographical origin in the cases of traceability of mango produce. Varietal signature and geographical traceability have high relevance in long term requirement to protect variety sovereignty along with GI protection, if granted or needed. Our genomic resources can be used as a research tool for development of varietal signature of targeted varieties. *MiSatDb* genomic resources provides a list of common SNPs across selected varieties which can be multiplexed for varietal differentiation. Our database also provides haplotype which can also be compared across varieties in varietal signature development. Such limited varietal differentiation in mango varieties viz., Neelam, Dashehari and Amrapali is recently reported using SNPs^53^.

RAD genomic resources based limited number of SNP has been used in trait improvement by cost-effective marker development tagging a disease resistance gene in molecular breeding of crop^54^. RAD data has been of immense use in species lacking sufficient genomic resources including reference sequence. In this situation, it has great advantage of low per-sample cost needed to generate millions of molecular markers required for genomic selection^55^. RAD data has been used for population structure and degree of admixture analysis. Such analysis has even revealed footprint of domestication and bottleneck^56^ as well as climate change events^57^ in crops.

Even after availability of mango whole genome reference sequence in future, the present genomic resources can be still of immense value. These SNPs can be mapped over mango genome reference sequence to obtain their exact physical location on each and every chromosome, thus obviating the need of re-sequencing to get similar information. Obtaining such genomic co-ordinates of each SNP will have further additional advantage to select them at equal spacing in order to minimise number of SNPs (with respect to each haplotype) without compromising the efficacy of genomic selection in future. Such approach even reduces the cost of genotyping. Use of RAD SNPs over existing ref-sequence has been very successfully used to enrich variety specific genomic resources of crop like rice with rapidity and cost effectiveness along with reduced computational complexities^25^.

## Conclusion

From a total of 84 mango varieties, 1.25 M SNPs using 171.80786 M RAD sequences are reported and catalogued in web genomic resource *MiSNPDb*. 749 SNPs were common to all these 84 varieties, which were used for diversity analysis and phylogeography studies. *MiSNPDb* has customized advance search options for haplotypes, depth and the varieties having common SNPs. The average genome coverage was 8.36% and average SNP depth was found to be 9.815. These resources can be used in mapping, QTL discovery, varietal differentiation, traceability, genome finishing and SNP array development in genetic improvement program and management of mango germplasm. Our study reveals that diversity of both soil and tropical ecology have contributed to evolution and differentiation of Indian mango varieties having a unique appearance, taste and aroma in different geographical regions.

## Methods

### Mango genomic DNA extraction and ddRAD-sequencing

High-molecular weight DNA from 91 leaf tissue samples was extracted and prepared for sequencing according to the modified version of RAD sequencing is known as Double Digestion Restriction site Associated DNA sequencing (ddRAD-Seq)^58^. One μg DNA from each sample was digested with *Sph*l and *Mluc*1 restriction enzyme and purified using AMPure XP Beads (Beckman Coulter, USA). Using T_4_ DNA ligase ligation of barcoded P_1_ and P_2_ adaptors was done and size selection of the product was done after 2% agarose gel electrophoresis. PCR amplification was done to enrich and add the Illumina specific adapters and flow cell annealing sequence, final quality check was done on bio analyzer. Sequencing was performed using Illumina TrueSeq chemistry (2X100nt) on Illumina HiSeq 2000 platform.

### Preprocessing of ddRAD-Seq data

The ddRAD-Seq data of 84 varieties of mango were preprocessed using the FASTX-Toolkit^59^. The number of sequences before quality trimming were 177395296 with a total of 17898214358 bases. In order to remove low quality sequence data, the data was quality trimmed using the fastq_quality_trimmer command of the FASTX-Toolkit. The minimum phred-score of 30 and minimum sequence length of 30 base pairs was used as a threshold for quality trimming.

### *De novo* SNP mining

*De novo* SNP mining was done by tool STACKS^60^ software version 1.29 using processed paired end RAD-Seq data of 84 varieties of mango. The *denovo_map.pl* perl wrapper script of the tool was used having three components *viz*., *ustacks, cstacks and sstacks* using the standard parameters. Population genetics statistics and transition and transversion ratio were calculated using in house perl script.

### Diversity analysis

Genetic diversity in the 84 varieties of mango was estimated using major and minor allele frequency, observed and expected heterozygosity as well as observed and expected homozygosity, and nucleotide diversity (π) at each locus. These parameters were calculated using the population program of the stacks pipeline. The population genetic statistics were generated using the population program of stacks pipeline using the common SNPs found in all the 84 varieties. Following the SNP mining and population genetic statistics, phylogenetic tree was drawn using the phylip file generated by the population program of the STACKS based on all the samples.

### Construction of phylogenetic tree

The SNPs from 84 varieties of mango mined using stacks software using the *denovo_map.pl* and population program which can give the results in vcf format and phylip format. The SNPs common in 84 varieties of mango varieties were retrieved from the vcf file constructed by the stacks software. The number of common SNPs is 749 out of 1192 SNPs. The phylip tree of the common SNPs was constructed by PHyML software version 3.0^61^ using BioNJ (neighbor joining) algorithm. The phylogenetic tree constructed by the PHyML software was visualized using the software *Figtree* version 1.4.0^62^.

### The Database Development

*MiSNPdb* (*Mangifera indica* SNP database), a relational database with *three-tier architecture* was developed using PHP, MySQL and Javascript. These three tiers are client, middle and database tier. Various web pages are developed for database browsing. The queries by users are placed in client tier. Database includes tables for SNPs from different mango varieties. In order to fetch the data from database and execute the query fired by the user, middle tier has a role in server side scripting using PHP. The database is freely accessible at the http://webtom.cabgrid.res.in/mangosnps/.

## Electronic supplementary material

Supplementary Tables
