# Supplementary material for: MiSNPDb: a web-based genomic resources of tropical ecology fruit mango (Mangifera indica L.) for phylogeography and varietal differentiation
Source: Sci Rep. 2017 Nov 2;7:14968. doi: 10.1038/s41598-017-14998-2 (PMC5668432; doi:10.1038/s41598-017-14998-2)
Supplement: Supplementary file 1 — Supplementary Tables [file 41598_2017_14998_MOESM1_ESM.doc]

***MiSNPDb*: a web based genomic resources of tropical ecology fruit mango (*Mangifera indica* L.) for phylogeography and varietal differentiation**

MA Iquebal^1^, Sarika Jaiswal^1^, Ajay Kumar Mahato^2^, Pawan K. Jayaswal^2^, UB Angadi^1^, Neeraj Kumar^1^, Nimisha Sharma^3^, Anand K. Singh^3^, Manish Srivastava^3^, Jai Prakash^3^, SK Singh^3^, Kasim Khan^4^, Rupesh K. Mishra^4^, Shailendra Rajan^4^, Anju Bajpai^4^, BS Sandhya^5^, Puttaraju Nischita^5^, KV Ravishankar^5^, MR Dinesh^5^, Anil Rai^1^, Dinesh Kumar^1,*^, Tilak R. Sharma^2^ and Nagendra K. Singh^2^

^1^. Centre for Agricultural Bioinformatics, ICAR-IASRI, New Delhi, India

^2^. ICAR-National Research Centre on Plant Biotechnology, New Delhi, India

^3^. ICAR-Indian Agricultural Research Institute, New Delhi, India

^4^. ICAR-Central Institute for Subtropical Horticulture, Lucknow, India

^5^. ICAR-Indian Institute of Horticultural Research, Bengaluru, India

**Supplementary Table 1. The RAD genomic data details of raw data of 84 mango varieties**

|  | **Variety Name** | **Number of Sequences** | **Number of Bases** |
| --- | --- | --- | --- |
|  | Afeam | 1780916 | 179872516 |
|  | Alphan | 3663356 | 368985790 |
|  | Alphanso | 1570856 | 158656456 |
|  | Amin Prince | 1767810 | 178548810 |
|  | Amrapali | 1718862 | 173169958 |
|  | Arka Aruna | 1965006 | 198465606 |
|  | Arunika | 1773500 | 178035842 |
|  | Baganapalli | 2433328 | 245758940 |
|  | Banganpalli | 2250674 | 227318074 |
|  | Banglora | 1357274 | 137084674 |
|  | Baramasi | 4131518 | 417283318 |
|  | Baramasi Ajholi | 1888612 | 190749812 |
|  | Bathui | 1423266 | 143749866 |
|  | Bhadaiya Sukul | 1868382 | 188706582 |
|  | Bhadauran | 2463130 | 248776130 |
|  | Bombay | 761198 | 76880998 |
|  | Bombay Green | 1614146 | 163028746 |
|  | Bombay Yellow | 1117748 | 112892548 |
|  | Bride of Russia | 4529590 | 457488590 |
|  | Carabao | 1740118 | 175751918 |
|  | Chandrakaran | 2082776 | 210360376 |
|  | Chinku | 1942150 | 194255030 |
|  | Creeping II | 2357426 | 237761046 |
|  | Dushehari | 2200984 | 222299384 |
|  | Edward | 1628482 | 164476682 |
|  | Elaichi | 842098 | 85051898 |
|  | Extrema | 2367784 | 238358808 |
|  | Fazri | 1625236 | 164148836 |
|  | Fazri Kalam | 1218364 | 123054764 |
|  | Gilas | 766000 | 77366000 |
|  | Gola Bhadaiya | 4204702 | 424349784 |
|  | Gourjeet | 848410 | 85689410 |
|  | Gulab Khas Green | 1412346 | 142646946 |
|  | Hardil Aziz | 1318446 | 133163046 |
|  | Heraswania | 4325306 | 436846200 |
|  | Himsagar | 2829140 | 285743140 |
|  | Hyb. 165 | 3774572 | 379590710 |
|  | Irwin | 4219684 | 424478930 |
|  | Iturba | 2298528 | 232151328 |
|  | Janardan Pasand | 2542954 | 256838354 |
|  | Kala | 2549276 | 257476876 |
|  | Kalapahar | 961802 | 97142002 |
|  | Karishad | 890116 | 89901716 |
|  | Kesar | 2275194 | 229794594 |
|  | Khasulkhas | 1837998 | 185637798 |
|  | Kothapalli Kobbari | 2302198 | 232521998 |
|  | Kurukkan | 1185782 | 119763982 |
|  | Langra | 3022664 | 305289064 |
|  | Langra Gorakhpur | 1532870 | 154819870 |
|  | Machhli | 3008696 | 303878296 |
|  | Malda | 3027112 | 302800062 |
|  | Malihabad Safeda | 1693492 | 171042692 |
|  | Mallika | 2016062 | 203622262 |
|  | Manipur dwarf | 1505948 | 150637000 |
|  | Manorajan | 4540944 | 458635344 |
|  | Mohammada Vikarabad | 4856946 | 490551546 |
|  | Mohanbhog | 1747334 | 174781550 |
|  | Mombosa | 3118020 | 314920020 |
|  | Mulgoa | 1138464 | 114984864 |
|  | Mundappa Black | 819138 | 82732938 |
|  | Neelum | 2238612 | 225824988 |
|  | Nekkare | 2441646 | 245475628 |
|  | Prabhashankar | 530724 | 53128494 |
|  | Primor de Amoreira | 828558 | 83684358 |
|  | Pusa Arunima | 3569438 | 360513238 |
|  | Pusa Lalima | 1745962 | 176342162 |
|  | Pusa Peetamber | 2972330 | 299077210 |
|  | Pusa Pratibha | 999854 | 100985254 |
|  | Pusa Shersth | 1295402 | 130835602 |
|  | Pusa Surya | 1897536 | 191651136 |
|  | Ramkela | 1633864 | 165020264 |
|  | Rataul | 3331438 | 336475238 |
|  | Ratna | 1433150 | 144748150 |
|  | Rosari | 1890318 | 190922118 |
|  | Safdar Pasand | 1548456 | 156394056 |
|  | Samar Bahist Alibagh | 840124 | 84852524 |
|  | Seipia | 2693750 | 272068750 |
|  | Sensation | 1255280 | 126783280 |
|  | Sonatol | 912592 | 92171792 |
|  | Sukul | 1533684 | 154902084 |
|  | Suvarnarekha | 5657662 | 571423862 |
|  | Tatoul | 411220 | 41188688 |
|  | Willard | 4550838 | 459634638 |
|  | Zardalu | 528124 | 53340524 |
| **Total** | | 177395296 | 17898214358 |

**Supplementary Table 2. Population diversity analysis statistics of 84 mango varieties**

| **Population** | **Private alleles** | **Sites** | **Variant Sites** | **Polymorphic Sites** | **% Polymorphic Loci** | **Observed Heterozygosity** | **Observed Homozygosity** | **Expected Heterozygosity** | **Expected Homozygosity** | 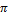 |
| --- | --- | --- | --- | --- | --- | --- | --- | --- | --- | --- |
| Langra | 0 | 66049 | 1170 | 211 | 0.3195 | 0.1803 | 0.8197 | 0.0902 | 0.9098 | 0.1803 |
| Himsagar | 3 | 66026 | 1149 | 194 | 0.2938 | 0.1688 | 0.8312 | 0.0844 | 0.9156 | 0.1688 |
| Heraswania | 0 | 66056 | 1177 | 143 | 0.2165 | 0.1215 | 0.8785 | 0.0607 | 0.9393 | 0.1215 |
| Sukul | 6 | 66046 | 1181 | 178 | 0.2695 | 0.1507 | 0.8493 | 0.0754 | 0.9246 | 0.1507 |
| Seipia | 6 | 66067 | 1188 | 140 | 0.2119 | 0.1178 | 0.8822 | 0.0589 | 0.9411 | 0.1178 |
| Tatoul | 0 | 65453 | 1143 | 75 | 0.1146 | 0.0656 | 0.9344 | 0.0328 | 0.9672 | 0.0656 |
| Prabhashankar | 2 | 65275 | 1121 | 122 | 0.1869 | 0.1088 | 0.8912 | 0.0544 | 0.9456 | 0.1088 |
| Mohanbhog | 0 | 65606 | 1188 | 112 | 0.1707 | 0.0943 | 0.9057 | 0.0471 | 0.9529 | 0.0943 |
| Manipur dwarf | 0 | 65544 | 1181 | 166 | 0.2533 | 0.1406 | 0.8594 | 0.0703 | 0.9297 | 0.1406 |
| Chinku | 0 | 65534 | 1185 | 171 | 0.2609 | 0.1443 | 0.8557 | 0.0722 | 0.9278 | 0.1443 |
| Malda | 0 | 65727 | 1188 | 104 | 0.1582 | 0.0875 | 0.9125 | 0.0438 | 0.9562 | 0.0875 |
| Kesar | 1 | 66069 | 1190 | 143 | 0.2164 | 0.1202 | 0.8798 | 0.0601 | 0.9399 | 0.1202 |
| Arka Aruna | 0 | 66067 | 1189 | 147 | 0.2225 | 0.1236 | 0.8764 | 0.0618 | 0.9382 | 0.1236 |
| Banglora | 2 | 66047 | 1184 | 172 | 0.2604 | 0.1453 | 0.8547 | 0.0726 | 0.9274 | 0.1453 |
| Baganapalli | 0 | 66063 | 1186 | 192 | 0.2906 | 0.1619 | 0.8381 | 0.0809 | 0.9191 | 0.1619 |
| Bride of Russia | 9 | 66070 | 1191 | 128 | 0.1937 | 0.1075 | 0.8925 | 0.0537 | 0.9463 | 0.1075 |
| Arunika | 1 | 65918 | 1182 | 149 | 0.226 | 0.1261 | 0.8739 | 0.063 | 0.937 | 0.1261 |
| Mallika | 0 | 66062 | 1188 | 180 | 0.2725 | 0.1515 | 0.8485 | 0.0758 | 0.9242 | 0.1515 |
| Afeam | 6 | 66068 | 1190 | 191 | 0.2891 | 0.1605 | 0.8395 | 0.0803 | 0.9197 | 0.1605 |
| Amin Prince | 1 | 66058 | 1188 | 135 | 0.2044 | 0.1136 | 0.8864 | 0.0568 | 0.9432 | 0.1136 |
| Bombay Yellow | 0 | 66010 | 1187 | 102 | 0.1545 | 0.0859 | 0.9141 | 0.043 | 0.957 | 0.0859 |
| Bombay | 0 | 65924 | 1176 | 93 | 0.1411 | 0.0791 | 0.9209 | 0.0395 | 0.9605 | 0.0791 |
| Bhadaiya Sukul | 4 | 66067 | 1190 | 124 | 0.1877 | 0.1042 | 0.8958 | 0.0521 | 0.9479 | 0.1042 |
| Bathui | 3 | 66046 | 1188 | 116 | 0.1756 | 0.0976 | 0.9024 | 0.0488 | 0.9512 | 0.0976 |
| Baramasi | 5 | 66070 | 1191 | 130 | 0.1968 | 0.1092 | 0.8908 | 0.0546 | 0.9454 | 0.1092 |
| Baramasi Ajholi | 2 | 66063 | 1190 | 114 | 0.1726 | 0.0958 | 0.9042 | 0.0479 | 0.9521 | 0.0958 |
| Gourjeet | 9 | 65943 | 1174 | 130 | 0.1971 | 0.1107 | 0.8893 | 0.0554 | 0.9446 | 0.1107 |
| Gola Bhadaiya | 5 | 66012 | 1189 | 139 | 0.2106 | 0.1169 | 0.8831 | 0.0585 | 0.9415 | 0.1169 |
| Gilas | 3 | 65911 | 1169 | 94 | 0.1426 | 0.0804 | 0.9196 | 0.0402 | 0.9598 | 0.0804 |
| Fazri Kalam | 0 | 66035 | 1184 | 131 | 0.1984 | 0.1106 | 0.8894 | 0.0553 | 0.9447 | 0.1106 |
| Fazri | 0 | 66051 | 1183 | 130 | 0.1968 | 0.1099 | 0.8901 | 0.0549 | 0.9451 | 0.1099 |
| Creeping II | 2 | 65971 | 1186 | 134 | 0.2031 | 0.113 | 0.887 | 0.0565 | 0.9435 | 0.113 |
| Langra Gorakhpur | 1 | 66052 | 1182 | 117 | 0.1771 | 0.099 | 0.901 | 0.0495 | 0.9505 | 0.099 |
| Kalapahar | 0 | 65937 | 1173 | 124 | 0.1881 | 0.1057 | 0.8943 | 0.0529 | 0.9471 | 0.1057 |
| Malihabad Safeda | 2 | 66057 | 1190 | 131 | 0.1983 | 0.1101 | 0.8899 | 0.055 | 0.945 | 0.1101 |
| Carabao | 0 | 66069 | 1191 | 190 | 0.2876 | 0.1595 | 0.8405 | 0.0798 | 0.9202 | 0.1595 |
| Primor de Amoreira | 5 | 65865 | 1166 | 176 | 0.2672 | 0.1509 | 0.8491 | 0.0755 | 0.9245 | 0.1509 |
| Janardan Pasand | 0 | 66060 | 1185 | 135 | 0.2044 | 0.1139 | 0.8861 | 0.057 | 0.943 | 0.1139 |
| Zardalu | 8 | 65737 | 1151 | 99 | 0.1506 | 0.086 | 0.914 | 0.043 | 0.957 | 0.086 |
| Bombay Green | 1 | 66040 | 1186 | 106 | 0.1605 | 0.0894 | 0.9106 | 0.0447 | 0.9553 | 0.0894 |
| Kala | 0 | 66059 | 1190 | 117 | 0.1771 | 0.0983 | 0.9017 | 0.0492 | 0.9508 | 0.0983 |
| Alphan | 0 | 65978 | 1190 | 118 | 0.1788 | 0.0992 | 0.9008 | 0.0496 | 0.9504 | 0.0992 |
| Mombosa | 0 | 66069 | 1190 | 109 | 0.165 | 0.0916 | 0.9084 | 0.0458 | 0.9542 | 0.0916 |
| Machhli | 0 | 66067 | 1188 | 141 | 0.2134 | 0.1187 | 0.8813 | 0.0593 | 0.9407 | 0.1187 |
| Edward | 8 | 66030 | 1189 | 160 | 0.2423 | 0.1346 | 0.8654 | 0.0673 | 0.9327 | 0.1346 |
| Hardil Aziz | 0 | 65990 | 1179 | 127 | 0.1925 | 0.1077 | 0.8923 | 0.0539 | 0.9461 | 0.1077 |
| Gulab Khas Green | 3 | 66020 | 1178 | 117 | 0.1772 | 0.0993 | 0.9007 | 0.0497 | 0.9503 | 0.0993 |
| Khasulkhas | 1 | 66050 | 1187 | 127 | 0.1923 | 0.107 | 0.893 | 0.0535 | 0.9465 | 0.107 |
| Sonatol | 1 | 65924 | 1160 | 132 | 0.2002 | 0.1138 | 0.8862 | 0.0569 | 0.9431 | 0.1138 |
| Samar Bahist Alibagh | 1 | 65877 | 1164 | 104 | 0.1579 | 0.0893 | 0.9107 | 0.0447 | 0.9553 | 0.0893 |
| Alphanso | 2 | 66053 | 1190 | 109 | 0.165 | 0.0916 | 0.9084 | 0.0458 | 0.9542 | 0.0916 |
| Amrapali | 0 | 65959 | 1190 | 165 | 0.2502 | 0.1387 | 0.8613 | 0.0693 | 0.9307 | 0.1387 |
| Dushehari | 0 | 66062 | 1189 | 106 | 0.1605 | 0.0892 | 0.9108 | 0.0446 | 0.9554 | 0.0892 |
| Pusa Surya | 0 | 66042 | 1189 | 165 | 0.2498 | 0.1388 | 0.8612 | 0.0694 | 0.9306 | 0.1388 |
| Neelum | 0 | 65999 | 1190 | 175 | 0.2652 | 0.1471 | 0.8529 | 0.0735 | 0.9265 | 0.1471 |
| Pusa Arunima | 0 | 66070 | 1191 | 158 | 0.2391 | 0.1327 | 0.8673 | 0.0663 | 0.9337 | 0.1327 |
| Pusa Lalima | 0 | 66060 | 1187 | 156 | 0.2361 | 0.1314 | 0.8686 | 0.0657 | 0.9343 | 0.1314 |
| Pusa Pratibha | 0 | 65986 | 1176 | 157 | 0.2379 | 0.1335 | 0.8665 | 0.0668 | 0.9332 | 0.1335 |
| Pusa Shersth | 0 | 66036 | 1180 | 155 | 0.2347 | 0.1314 | 0.8686 | 0.0657 | 0.9343 | 0.1314 |
| Rataul | 0 | 66070 | 1191 | 116 | 0.1756 | 0.0974 | 0.9026 | 0.0487 | 0.9513 | 0.0974 |
| Extrema | 4 | 65977 | 1189 | 194 | 0.294 | 0.1632 | 0.8368 | 0.0816 | 0.9184 | 0.1632 |
| Hyb. 165 | 0 | 65979 | 1191 | 189 | 0.2865 | 0.1587 | 0.8413 | 0.0793 | 0.9207 | 0.1587 |
| Irwin | 0 | 65981 | 1191 | 110 | 0.1667 | 0.0924 | 0.9076 | 0.0462 | 0.9538 | 0.0924 |
| Iturba | 19 | 66054 | 1188 | 207 | 0.3134 | 0.1742 | 0.8258 | 0.0871 | 0.9129 | 0.1742 |
| Kurukkan | 14 | 65940 | 1172 | 181 | 0.2745 | 0.1544 | 0.8456 | 0.0772 | 0.9228 | 0.1544 |
| Safdar Pasand | 0 | 66039 | 1181 | 150 | 0.2271 | 0.127 | 0.873 | 0.0635 | 0.9365 | 0.127 |
| Ramkela | 0 | 65981 | 1141 | 135 | 0.2046 | 0.1183 | 0.8817 | 0.0592 | 0.9408 | 0.1183 |
| Rosari | 0 | 66015 | 1154 | 120 | 0.1818 | 0.104 | 0.896 | 0.052 | 0.948 | 0.104 |
| Pusa Peetamber | 0 | 65981 | 1190 | 165 | 0.2501 | 0.1387 | 0.8613 | 0.0693 | 0.9307 | 0.1387 |
| Ratna | 0 | 66043 | 1183 | 149 | 0.2256 | 0.126 | 0.874 | 0.063 | 0.937 | 0.126 |
| Sensation | 0 | 66005 | 1181 | 158 | 0.2394 | 0.1338 | 0.8662 | 0.0669 | 0.9331 | 0.1338 |
| Bhadauran | 4 | 66068 | 1191 | 69 | 0.1044 | 0.0579 | 0.9421 | 0.029 | 0.971 | 0.0579 |
| Banganpalli | 20 | 66068 | 1191 | 197 | 0.2982 | 0.1654 | 0.8346 | 0.0827 | 0.9173 | 0.1654 |
| Kothapalli Kobbari | 1 | 66054 | 1188 | 138 | 0.2089 | 0.1162 | 0.8838 | 0.0581 | 0.9419 | 0.1162 |
| Karishad | 0 | 65941 | 1176 | 135 | 0.2047 | 0.1148 | 0.8852 | 0.0574 | 0.9426 | 0.1148 |
| Elaichi | 13 | 65941 | 1169 | 148 | 0.2244 | 0.1266 | 0.8734 | 0.0633 | 0.9367 | 0.1266 |
| Chandrakaran | 5 | 66050 | 1185 | 124 | 0.1877 | 0.1046 | 0.8954 | 0.0523 | 0.9477 | 0.1046 |
| Mohammada Vikarabad | 10 | 66064 | 1188 | 173 | 0.2619 | 0.1456 | 0.8544 | 0.0728 | 0.9272 | 0.1456 |
| Suvarnarekha | 33 | 66057 | 1178 | 312 | 0.4723 | 0.2649 | 0.7351 | 0.1324 | 0.8676 | 0.2649 |
| Willard | 9 | 66058 | 1179 | 182 | 0.2755 | 0.1544 | 0.8456 | 0.0772 | 0.9228 | 0.1544 |
| Manorajan | 10 | 66054 | 1176 | 255 | 0.386 | 0.2168 | 0.7832 | 0.1084 | 0.8916 | 0.2168 |
| Mulgoa | 2 | 65947 | 1134 | 220 | 0.3336 | 0.194 | 0.806 | 0.097 | 0.903 | 0.194 |
| Nekkare | 4 | 65971 | 1186 | 185 | 0.2804 | 0.156 | 0.844 | 0.078 | 0.922 | 0.156 |
| Mundappa Black | 27 | 65911 | 1150 | 173 | 0.2625 | 0.1504 | 0.8496 | 0.0752 | 0.9248 | 0.1504 |

**Supplementary Table 3.** List of mango varieties with phylogenetic cluster and geographical distribution*

| **S.No** | **Variety** | **Phylogenetic Cluster** | **Geographical Distribution** | **Latitude and Longitude** |
| --- | --- | --- | --- | --- |
|  | Langra | Group II/East variety | Bihar | 25.09°N 85.31°E |
|  | Himasagar | Group II/East Variety | West Bengal | 22.98° N, 87.85° E |
|  | Herswania | Group II/East Variety | West Bengal | 22.98° N, 87.85° E |
|  | Sukul | Group IV/Hybrid/Exotic Variety | Bihar | 25.09°N 85.31°E |
|  | Seipia | Group II/East Variety | Bihar | 25.09°N 85.31°E |
|  | Tatoul | Group I/North Variety | No literature available |  |
|  | Prabhashankar | Group II/East Variety | Bihar | 25.09°N 85.31°E |
|  | MohanBhog | Group II/East Variety | Bihar | 25.09°N 85.31°E |
|  | Manipur Dwarf | Group IV/South variety | Manipur | 24.66° N, 93.90° E |
|  | Chinku | Group IV/South variety | No literature available |  |
|  | Malda | Group II/East Variety | West Bengal | 22.98° N, 87.85° E |
|  | Kesar | Group III/South variety | Gujarat/Maharashtra | 19.75° N, 75.71° E |
|  | Arka Aruna | Group IV/Hybrid Variety | Karnataka | 15.31° N, 75.71° E |
|  | Bangalora | Group III/South Varity | Tamil Nadu | 11.12° N, 78.65° E |
|  | Banganpalli | Group I/North Variety | Andhra Pradesh | 15.91° N, 79.74° E |
|  | Bride of Russia | Group II/East Variety | Uttar Pradesh | 26.84° N, 80.94° E |
|  | Dushehari | Group I/North Varity | Uttar Pradesh | 26.84° N, 80.94° E |
|  | Arunika  (Amrapalli*Vanraj) | Group IV/Hybrid Variety | Hybrid variety |  |
|  | Mallika  (Neelum*Dushehari) | Group III/South Varity | Neelum is indigenous to Tamil Nadu | 11.12° N, 78.65° E |
|  | Afeam | Group III/South variety | West Bengal | 22.98° N, 87.85° E |
|  | Amin Prince (also known as Amin Tehsil) | Group I/North Variety | Uttar Pradesh | 26.84° N, 80.94° E |
|  | Bombay Yellow | Group II/East Variety | West Bengal | 22.98° N, 87.85° E |
|  | Bombai | Group II/East Variety | Bihar | 25.09°N 85.31°E |
|  | Bhadaiya Sukul | Group II/East Variety | Bihar | 25.09°N 85.31°E |
|  | Bathui | Group I/North Variety | Bihar | 25.09°N 85.31°E |
|  | Baramasi | Group I/North Variety | Uttar Pradesh | 26.84° N, 80.94° E |
|  | Baramasi Ajholi | Group I/North Variety | Uttar Pradesh | 26.84° N, 80.94° E |
|  | Gourjeet | Group I/North Variety | No literature available |  |
|  | Gola bhadiya | Group I/North Variety | Uttar Pradesh | 26.84° N, 80.94° E |
|  | Gilas | Group I/North Variety | Uttar Pradesh | 26.84° N, 80.94° E |
|  | Fazri Kalam | Group I/North Variety | Uttar Pradesh | 26.84° N, 80.94° E |
|  | Fazri | Group I/North Variety | Bihar | 25.09°N 85.31°E |
|  | Creeping II | Group I/North Variety | Kerala | 10.8505° N, 76.2711° E |
|  | Langra Gorakhpur | Group I/North Variety | Uttar Pradesh | 26.84° N, 80.94° E |
|  | Kalapahar | Group II/East Variety | West Bengal | 22.98° N, 87.85° E |
|  | Malihabad Sefada | Group I/North Variety | Uttar Pradesh | 26.84° N, 80.94° E |
|  | Carabao | Group IV/Exotic Variety | Philippines | 12.87° N, 121.77° E |
|  | Primor de Amoreiria | Group I/North Variety | Kerala | 10.8505° N, 76.2711° E |
|  | Janardhan Pasand | Group III/South Variety | Andhra Pradesh | 15.91° N, 79.74° E |
|  | Zardalu | Group I/North Variety | West Bengal | 22.98° N, 87.85° E |
|  | Bombay Green | Group II/East Variety | Uttar Pradesh | 26.84° N, 80.94° E |
|  | Kala | Group II/East Variety | No literature available |  |
|  | Alphan | Group II/East Variety | No literature available |  |
|  | Mombosa | Group II/East Variety | No literature available |  |
|  | Machli | Group II/East Variety | Uttar Pradesh | 26.84° N, 80.94° E |
|  | Edward | Group IV/Hybrid/Exotic | South Florida (USA) | 27.6648° N, 81.5158° W |
|  | Hardil Aziz | Group I/North Variety | Uttar Pradesh | 26.84° N, 80.94° E |
|  | Gulab khas green | Group II/East Variety | Odisha | 20.9517° N, 85.0985° E |
|  | Khas ul khas | Group II/East Variety | Uttar Pradesh | 26.84° N, 80.94° E |
|  | Sonatol | Group III/South variety | No literature available |  |
|  | Samar bahist Alibagh | Group II/East Variety | Bihar | 25.09°N 85.31°E |
|  | Alphonso | Group III/South Variety | Maharashtra/Goa/TamilNadu/Karnatka | 19.75° N, 75.71° E |
|  | Amrapalli | Group III/South Variety | Delhi | 28.7041° N, 77.1025° E |
|  | Pusa Surya | Group IV/Hybrid/Exotic Variety | Exotic Eldon variety introduced from Brazil | 14.23° S, 51.92° W |
|  | Neelum | Group III/South Variety | Tamil Nadu | 11.12° N, 78.65° E |
|  | Pusa Arunima (Amrapalli*Sensation) | Group III/South Variety | Hybrid variety |  |
|  | Pusa Lalima  (Dasehari*Sensation) | Group I/North Variety | Hybrid variety |  |
|  | Pusa Pratibha  (Amrapalli*Sensation) | Group III/South Variety | Hybrid variety |  |
|  | Pusa Shresth  (Amrapalli*Sensation) | Group III/South Variety | Hybrid variety |  |
|  | Rataul | Group I/North Variety | Uttar Pradesh | 26.84° N, 80.94° E |
|  | Extrema | Group I/North Variety | Exotic Variety |  |
|  | Hybrid 165 | Group III/South variety | No literature available |  |
|  | Irwin  (Lipens*Haden) | Group IV/Hybrid/Exotic Variety | South Florida (USA) | 27.6648° N, 81.5158° W |
|  | Iturba | Group I/North variety | Exotic Variety |  |
|  | Kurukkan | Group III/South Variety | Kerala | 10.8505° N, 76.2711° E |
|  | Safdar Pasand | Group I/North Variety | West Bengal | 22.98° N, 87.85° E |
|  | Ram kela | Hybrid/Exotic Variety | Punjab | 31.1471° N, 75.3412° E |
|  | Rosari | Hybrid/Exotic Variety | Exotic Variety |  |
|  | Pusa peetamber  (Amrapalli*Lal sundary) | Group I/North Variety | Hybrid Variety |  |
|  | Willard | Group IV/Hybrid/Exotic Variety | Exotic Variety from Srilanka | 7.87° N, 80.77° E |
|  | Ratna  (Neelum*Alphonso) | Group III/South variety | Maharashtra/Tamil Nadu  (Neelum is indigenous to Tamil Nadu) | 11.12° N, 78.65° E |
|  | Sensation | Hybrid/Exotic Variety | Exotic Variety from South Florida | 27.6648° N, 81.5158° W |
|  | Bhadauran | Group I/North Variety | Uttar Pradesh | 26.84° N, 80.94° E |
|  | Kothapalli Kobari | Group III/South Varity | Andhra Pradesh | 15.91° N, 79.74° E |
|  | Karishad | Group IV/Hybrid/Exotic | Karnataka | 15.31° N, 75.71° E |
|  | Elaichi | Group IV/Hybrid/Exotic Variety | Andhra Pradesh | 15.91° N, 79.74° E |
|  | Chandrakaran | Group I/North Variety | Kerala | 10.8505° N, 76.2711° E |
|  | Mohamadda Vikarabad | Group III/South Varity | Andhra Pradesh | 15.91° N, 79.74° E |
|  | Suvaranrekha | Group III/South Varity | Andhra Pradesh | 15.91° N, 79.74° E |
|  | Manoranjan | Group III/South Varity | Madras Sate (present Tamil Nadu) | 11.12° N, 78.65° E |
|  | Mulgoa | Group III/South Varity | Tamil Nadu | 11.12° N, 78.65° E |
|  | Nekkare | Group III/South Varity | Karnataka | 15.31° N, 75.71° E |
|  | Baganpalli | Group III/South Varity | Karnataka | 15.31° N, 75.71° E |
|  | Mundappa Black | Group III/South Varity | Karnataka | 15.31° N, 75.71° E |

* Ravishankar *et al*., 2015 (Reference 37 in main text)
